# Perceived Barriers to Rural Elderly Women’s Health-Promoting Behaviors: An Ecological Perspective

**DOI:** 10.3390/ijerph17176107

**Published:** 2020-08-21

**Authors:** Hyunjung Moon, Sunkyung Cha, Eunyoung Park

**Affiliations:** 1 College of Nursing, Incheon Catholic University, Incheon 21987, Korea; hjmoon@iccu.ac.kr; 2 Department of Nursing Science, Sunmoon University, Asan 31460, Korea; skc0701@hanmail.net; 3 College of Nursing, Chungnam National University, Daejeon 35015, Korea

**Keywords:** rural elderly women, health-promoting behavior, ecological, barrier

## Abstract

This study multidimensionally examines rural elderly women’s subjective barriers to practicing health-promoting behaviors. Twenty-six rural elderly women participated in three focus group interviews. Content analysis and a qualitative research method were used. The results, based on an ecological model, show that the implementation of health-promoting behaviors in rural elderly women was comprehensively related to intrapersonal (functional decline, passive attitude, and lack of implementation), interpersonal (lack of social support), community (restrictive conditions, accessibility issues, and lack of infrastructure), and public policy (lack of policy support) factors. Interventions addressing each factor can help reduce or eliminate the perceived barriers to health-promoting behaviors through interactions. Our findings can contribute to the development of health-promoting programs focused specifically on the socialization of rural elderly women and community-centered health policies in the future.

## 1. Introduction

The aging global population is one of the most important social phenomena nowadays. The growing number of elderly people inevitably poses serious challenges to our society: as life expectancy increases, the risk of chronic diseases or injuries also increases. Chronic diseases related to functional decline or the collapse of social support systems reduce the well-being of the vulnerable elderly and cause excessive spending on care facilities and medical services [1,2]. Therefore, promoting the health of vulnerable elderly people who live in communities has attracted constant interest.

In Korea, the population of older adults aged 65 years or older comprises 14.9% of the total population; it is predicted that Korea will enter the post-aged society by 2026. The mean life expectancy of Korean men and women is 79.3 and 85.4 years, respectively, a 6.1-year difference. Moreover, women aged 65 years or older comprise 57.3% of the total elderly population. That is, much of the issues affecting the elderly may be issues that affect elderly women specifically [3]. In particular, rural elderly women experience a low quality of life due to financial difficulties, psychosocial isolation, and lack of social support. They can also have many diseases due to a lack of health-promoting activities [4].

Since the health dynamics of the elderly are related to an increased need for health management, the rapid aging of the global population can be expected to result in an increased demand for health management services. Given the greater burden and reduced capacity to treat diseases in isolated regions, this trend can be expected to be more pronounced in an environment that lacks resources necessary for health promotion activities, such as rural regions [5,6]. Mostly, vulnerable elderly people in such regions cite cost and transportation as barriers they face when accessing healthcare services. In particular, transportation can be an important issue for rural elderly people because various healthcare facilities are concentrated in cities that are substantially far from them. Furthermore, the lack of accessible health information due to functional decline (e.g., loss of vision or hearing) can also be a barrier to health care access [5].

The sub-factors associated with health-promoting behaviors that have the greatest impact on Korean rural elderly people’s life satisfaction were nutrition, diet, exercise, and activities [7]. Kim explained that greater scores on the implementation of health-promoting behaviors were found in rural elderly people because this population tended to pursue such behaviors due to perceived poor health; therefore, in-depth studies on rural elderly people are necessary. Furthermore, residential environment can have an impact on health-promoting behaviors; for instance, there was a higher prevalence of depression among rural elderly people compared to those living in cities; however, rural elderly people exhibited good activities of daily living and subjective health [8]. A study that analyzed the social functions of older adults aged 65 years or older based on their physical location reported that the social functions of rural elderly people were lower than those of their urban counterparts, suggesting that this was a result of the social isolation experienced in rural regions [9]. Moreover, it was reported that the barrier to access home care services among elderly minorities was due to the lack of social networks to share and exchange information, along with language problems [10]. It can be inferred that this can also be caused by the regional gap between urban and rural areas. Ultimately, the main regional problems related to health-promoting behaviors in rural areas may be the limited healthcare services and lack of various healthcare infrastructures [6]; this can fundamentally be due to the physical characteristics of rural areas, which make it difficult to utilize community resources.

McLeroy’s ecological perspective combines the individual and environmental factors that affect behavior, and categorize them into intrapersonal-, interpersonal-, organizational-, community-, and public policy-level factors [11]. Intrapersonal factors include individual characteristics, such as knowledge, attitude, and behaviors, while interpersonal factors include formal or informal social networks and social support systems. Organizational factors refer to social institutions and rules of operation, community factors allude to organizations or institutions within the administrative district, and public policy factors include regional and national laws and policies [11].

Thus, the ecological perspective provides a useful conceptual framework to explore the social determinants of health in a community. The reason is that health-promoting behaviors are an extremely personal effort to achieve good health, including supplementing biological and genetic vulnerabilities and preventing diseases in advance, and physical and social environments can motivate these actions or not. For example, individuals’ health-promoting behaviors are vitalized when facilities for regular exercise are close by or when local government provides social health resources, such as health examination services or health-promoting programs [12,13]. In a qualitative study that analyzed social determinants of health based on ecological models for African Americans in rural areas, potential social determinants of health at a multidimensional ecological level were identified [14]. In a large-scale quantitative study on 4028 people which analyzed the relationship between perceived quality of life of the elderly and the living environment that supports the quality of life based on the ecological model, the perception of the environment explains 49% of the quality of life [15].

Numerous rural elderly individuals have limited access to health care and most of what we know about this problem is based on quantitative research. It is known that qualitative research can provide additional insight into individual perceptions about approaches to health care. Overall, rural elderly people can face diverse barriers to access necessary health management activities or programs. Through a qualitative study, rural elderly people’s perspectives can be understood. By providing a forum for elderly people to express their concerns about the current health care delivery system, information that is useful to create policies can be provided [16].

Understanding the factors that impact health-promoting behaviors from the ecological theory perspective and identifying the perceived barriers associated with them in community populations may therefore help the development of more comprehensive and effective interventions or policies [17]. Thus, this study aims to identify the barriers to health-promoting behaviors perceived by rural elderly women in order to provide basic data for the development of health promotion programs customized for this population.

## 2. Materials and Methods

### 2.1. Design

A qualitative approach was used; data were collected via focus groups in order to perform an in-depth exploration of the perceived barriers to health-promoting behaviors among rural elderly women. The data were analyzed using content analysis. Focus group interviews were employed because they provide rich data in the research participants’ own words and lead to a synergistic effect through interactions with other participants in the group [18].

### 2.2. Participants

Participants were recruited via convenience sampling. The study sample consisted of 26 elderly women aged 65 years or older who lived in one of three villages in Y City (population of 111,525), located in the southeastern Gyeonggi Province, which is at the center of the Korean Peninsula [19]. Inclusion criteria were having lived in rural regions for at least one year, no disorders in communication, no psychiatric illnesses, and voluntary consent to participate.

### 2.3. Data Collection

The data for this study were collected from 1 to 30 May 2018. A total of three focus group interviews were conducted during this period. The researcher personally visited the senior center in each village with the help of the Medical Director in the region to explain the research aim and procedure. The focus group interviews were conducted at the senior center in each village in an acceptable, accessible, and stable atmosphere, as the research participants visited the senior centers almost every day. Chairs were placed in a circle so that all participants could see and hear each other. Snacks were served as it would help to promote communication. Each focus group interview lasted about 90 min. The interviews were hosted by the researcher who had experience moderating focus group interviews. The interviews started with a general description of the research to the participants and an introduction of the researcher, research assistant, and participants. Then, focus group interviews were conducted without predefined response options.

The focus group interviews started with opening questions, followed by introductory questions, transition questions, key questions, and ending questions [18], as shown in Table 1.

The interviews were recorded on two portable recorders; in addition to the recordings, the researcher and the research assistant took written notes of the on-site atmosphere and important discussion points that stood out. As the moderator, the researcher provided verbal and nonverbal messages to promote the dynamics and the interaction within the group and played the role of mediator and coordinator. The recorded contents of the interview were consistently transcribed by one undergraduate nursing student immediately after the interview. The transcription was verified by the researcher and research assistant who conducted the interview.

### 2.4. Ethical Considerations

This study was conducted after receiving approval from F University Institutional Review Board (No: FEUIRB-171130-01-3). The researchers described the research aim and procedure, recording of the interview contents, use of transcriptions, and guarantee of confidentiality to the participants who met the inclusion criteria. Written informed consent forms were obtained before participation. It was explained to the research participants that they could terminate the participation at any time and that all data would be anonymized to protect their privacy.

### 2.5. Data Analysis

The conceptual framework for analysis was based on the ecological theory. The transcription of each interview was analyzed through content analysis. The transcribed, raw materials were read repeatedly to extract meaningful sentences, and the following coding and categorization were performed to identify the main themes [20]:

- In order to identify the meaning, the data were labeled with the language expressed by the participants in the raw materials.
- Data were organized in order to derive the main themes.
- The themes were designated in a language that reflected the unique and detailed characteristics of the sample.
- By identifying the relationship between the identified themes and the four factors (intrapersonal-, interpersonal-, community-, and public policy-level factors) of the ecological theory, comparisons with and connections to other themes were made.

### 2.6. Rigor and Trustworthiness

To ensure the rigor and trustworthiness of our qualitative research, assessments were made according to the following four standards: credibility, fittingness, auditability, and confirmability [21]. First, to ensure credibility, the principal investigator (first author) and another author (corresponding author) reviewed the transcripts independently, before discussing the themes; discussions continued until there was an agreement about the extracted themes. Second, for fittingness, a portion of the participants were selected to check whether the extracted themes, subthemes, and the results of the study reflected what they expressed in order to review the accuracy of the results. Third, for auditability, it was checked whether each interview was systematically recorded and conducted through audited follow-ups, along with field notes and memos. Fourth, confirmability was ensured when the research process was conducted without any bias and the previous three standards were met.

## 3. Results

The general characteristics of the participants are shown in Table 2. The mean age of the participants was 78.8 years (range: 65–91). Regarding family arrangement, 42.3% lived with a spouse and 26.9% lived alone. Moreover, the majority of the participants had never attended school (53.8%) and had two or more chronic diseases (92.3%).

The results of this study were analyzed based on four out of the five factors proposed by the ecological theory; organizational factors were excluded as they were irrelevant in this study. The barriers that rural elderly women perceived to engage in health-promoting behaviors were identified as follows.

### 3.1. Intrapersonal Factors

#### 3.1.1. Functional Decline

Most (twenty) participants mentioned the impact of decline in physical functions (i.e., functional decline) on their ability to engage in health-promoting behaviors. Sensory dysfunctions, such as hearing or vision loss, memory decline, and pain were identified as hindering health-promoting behaviors. In particular, severe pain in the legs, back, and joints were the main barriers to movement.

*“I take three breaks to get here (senior center), because my back hurts (…) I work in the field, and my back hurts so I’m weak. It hurts everywhere, and it’s killing me (…) It is difficult because my body hurts. Even now, my back hurts. When I wake up in the morning, it hurts. It’s killing me.”*

(Participant 2–5)

Participants said that vigorous activities, which were previously possible, became impossible due to their lack of strength and energy and that they could not think about exercising for their health given that even walking had become difficult. In addition, ten participants referred to themselves as “general hospitals” because they had various health problems, including pain all over the body (e.g., headaches, leg pain), heartburn, and insomnia. Ultimately, participants believed that their inadequate physical functions were barriers to their health-promoting behaviors, such as doing exercises and physical activities.

*“It must’ve been a little over 25 years. I can’t walk because it hurts all of a sudden. I can’t even go this far. Now I’m trying to lose weight by 10 kg, but my knees hurt, too. I am in a lot of pain because of my spinal stenosis. I’m okay on the outside, but when I go to the hospital, even the university hospitals say ‘a big university general hospital.’ I can barely cook rice and go back and forth around here. I walked last year... but I couldn’t even sit down in 2003 because of the pain. It hurts when I’m just there, and it hurts when I’m lying down. It’s tense and it hurts.”*

(Participant 1–8)

#### 3.1.2. Passive Attitude

Participants were hesitant and cautious about active exercises as they experienced negative experiences of falling or tripping in the past that limited their physical activity. They avoided exercising because they thought they should not get hurt again from actively moving. They believed that the passive attitude resulting from these fears and worries was a barrier to their health-promoting behaviors.

*“I also fell on the floor and hurt my ankle and back. I would fall while going somewhere and trip while going somewhere else, so I got a surgery on my ankle. (…) I’m careful, because I can’t fall again. (…) I tripped while bringing my 4-year-old grandson, so now I’m careful (when I walk or move around since the fall). (Because I can’t fall again) since I’m weaker now. So, I should be careful.”*

(Participant 2–4)

Participants did not treat or manage their pain and also did not pay attention to it because they accepted pain as an inevitable part of aging. As a result of such perceptions, participants said that they neglected health-promoting behaviors, such as eating healthy food or exercising regularly, and became passive because they were lazy, not diligent, or had no will to exercise. They acknowledged their own lack of responsibility towards health-promoting behaviors.

*“Oh well, there’s no need to think about it too much. I am over 80 (years old) so I just eat what I eat (and don’t pay too much attention).”*

(Participant 2–2)

*“If I was more diligent, I can just walk around, but I’m lazy and don’t move much so that’s uncomfortable.”*

(Participant 2–3)

#### 3.1.3. Lack of Implementation

Participants mentioned that they did not exercise because it was not a habit for them, although they knew that it was necessary, citing excuses such as fatigue and pain as the reason they did not engage in health-promoting behaviors. They said that they gradually became disengaged from exercising because they did not enjoy it and did not want to participate in strenuous exercises. In other words, they expressed that they were aware of the need for maintaining their health, but this did not lead to steady practice.

*“If it’s too hard, I try not to do it... I can do exercises that I can do while sitting down. I only do things while sitting down. I won’t do them [exercises] while standing.”*

(Participant 1–9)

*“My doctor told me to do 100 repetitions of the leg exercises while lying down. I can’t do 100 repetitions, but I about 20–30 [when] I lie down every evening.”*

(Participant 2–2)

### 3.2. Interpersonal Factors: Interpersonal Processes and Primary Groups

#### Lack of Social Support

As barriers to health-promoting behaviors, participants expressed that they would engage in exercise or physical activities with others, but that they would not do so if they were alone. Moreover, it was difficult for the participants to actively engage in health-promoting behaviors even if they lived with their family. Even though they recognized that it was necessary to exercise, they perceived that not knowing how to do so was a problem, given that they did not get accurate and sufficient advice from people around them. Thus, for participants, the lack of a support system around them to engage in health-promoting behaviors as well as insufficient information were barriers to their health-promoting behaviors, in terms of interpersonal factors.

*“I really want to go out and do these exercises, and do yoga with you [other participants]. But none of old adults including me want to because we are all in pain. Really, I would if many people did it together. But no one does it at all.”*

(Participant 1–9)

*“[Exercising] is a bit difficult now. (Laughing) It is difficult even if I live with my daughter-in-law and grandson, because I am old.”*

(Participant 2–2)

### 3.3. Community Factors

#### 3.3.1. Restrictive Conditions

Participants explained that they were usually too busy and tired from farming, given the nature of the rural region; thus, it was hard to exercise unless it was off-season. They said that it was regrettable that there were unfavorable conditions associated with access to health care services, including having to walk to save money on the transportation costs associated with travel to and from the facilities or not using the facilities altogether because they were not financially well-off.

*“For me, it is difficult to go exercise at those places like gyms in the rural regions. I’m tired if I go out. I am too (tired) from farming. I have to rest when I can, but I don’t come back out. I farm a lot, and I only go out during off-season.”*

(Participant 1–9)

*“I go to the hospital often and get acupuncture because of my back. My back isn’t so bad that it needs surgery, but it needs consistent care. But is that possible in a rural village?”*

(Participant 2–8)

#### 3.3.2. Accessibility Issues

The public transportation needed to visit the medical institutions or exercise facilities was inconvenient; for participants, it was not easy to use public transportation with long service intervals. Moreover, walking to the exercise facilities was challenging because they were far from them and it took them long to get there. As such, they naturally became neglectful about exercising or participating in health-promoting activities.

*“I go on the bus. The bus schedule is not frequent. It comes every two hours. So, it’s very (difficult) to go to Janghowon (a place with relatively higher number of clinic-level medical facilities). It’s iffy, because the bus doesn’t come often. (…) But it’s hard to go because you have to walk there.”*

(Participant 2–4)

*“It’s inconvenient because I have to keep track of time to go (to the hospital). I have to wait two or three hours if I miss the bus.”*

(Participant 3–4)

#### 3.3.3. Lack of Infrastructure

Participants noted that the lack of infrastructure for health and lifestyle management in rural areas compared to cities was a barrier to consistent health management. They expressed not traveling far on purpose for health management and that there were no health care facilities near their homes. In particular, a participant who lived in a city until a few years ago noted that a significant shortage of exercise facilities or health care programs in rural areas compared to urban areas was a negative factor for health-promoting behaviors.

*“It has been less than 2 years since I moved (to the rural area). I came from the city. I love everything here, including the people that live in the neighborhood. It’s only that it’s the winter now, and I’m not able to engage in cultural activities. If I was more diligent, I can go (far by driving), but it’s inconvenient because I’m a bit lazy and I end up not going.”*

(Participant 2–3)

### 3.4. Public Policy

#### Lack of Policy Support

Participants complained that as the health care budget has been reduced, it is difficult to access healthcare products, such as drugs and heating pads, which were previously provided for free at the health clinic. They said they would like the city to allocate more funds to health services so that they could use them.

*“I would like it if I could get more. Before, public health clinics and health clinics made some money so I was able to use them, like drugs and products…. But now that the city is managing it, I think the budget is tight. I used to get heating pads when I visited. But now, there’s nothing like that.”*

(Participant 1–8)

## 4. Discussion

In order to identify the perceived barriers to health-promoting behaviors among elderly women living in rural regions, which are relatively vulnerable locations, this study directly listened to their opinions and analyzed such barriers from a multi-dimensional perspective, based on an ecological approach. Therefore, the results of this qualitative analysis, along with the existent quantitative data, provide evidence that can be applied to the development of an integrative health-promoting intervention program.

Our results indicate that a substantial proportion of what rural elderly women perceive to be barriers to health-promoting behaviors are associated with intrapersonal factors. Functional decline and pain were the major identified barriers to health-promoting behaviors, followed by passive attitudes due to previous experiences with falling/injuries. This is consistent with previous findings that physical illnesses and limitations impacted health-promoting behaviors negatively, along with lack of self-confidence [22]. In this study, rural elderly women perceived pain as inevitable with aging and, thus, accepted it. Consequently, they did not think that they needed special health management services. Considering the previous findings that perceptions about aging affect older adults’ quality of life [23,24], a change in such perceptions and attitudes toward health care is desirable. In this study, even older adults who knew that it was necessary to pursue health-promoting behaviors grew distant from exercising due to reasons such as hating strenuous exercise and feeling fatigue or pain. At the same time, some participants continued to exercise, but reduced the intensity to a suitable level. We would like to point out here that while the themes of passive attitude and lack of implementation sound similar, we have identified a clear distinction between them. We found that the practice of “not participating” in physical exercise, or avoiding it, was more of a behavioral problem. On the other hand, not engaging in health-promoting behaviors because they believed that certain health problems were a normal part of aging and therefore believing that it was okay to not do anything about the problems reflected a pattern of thinking and a passive attitude. Based on a study that positive attitudes do not necessarily lead to behavioral practice [25,26], we feel that it may be necessary to come up with and apply a psychosocial program that can bring about a positive change in the attitudes of the elderly, thereby leading to better health-promoting behaviors. In addition, for the elderly who cannot actually practice health promotion activities, a step-by-step approach may be useful from low intensity to gradually increasing intensity. According to previous research, to enhance elderly people’s participation in health-promoting activities, it is advised to customize routines depending upon health conditions [22,27], include health-promoting behaviors in religious activities [28], and establish a social circle that does not focus on health.

Regarding interpersonal barriers to health-promoting behaviors, participants generally did not know how to exercise, even though they knew that it was necessary, mainly because they did not get accurate and sufficient advice from those around them. Therefore, the lack of peers to exercise with and the lack of an accurate information system to guide health-promoting activities were the main interpersonal barriers identified. Elderly people need the support of their family because it is difficult for them to engage in health-promoting behaviors such as exercise by themselves [28]. In addition, older adults need to engage in health behaviors, such as physical activities, that are customized to their individual health condition [27]. Older people are more likely to rely on supportive resources, such as institutions and centers in the community, as their existing personal relationships are reduced due to the deaths of their spouses or friends and living apart from their children [9,29,30]. In particular, interpersonal support (e.g., forming support networks with their peers, relying on each other to improve physical activities and diet, or family members consistently providing positive feedback about health-promoting behaviors) is important in promoting desirable health behaviors in rural elderly people. Having social support can help with intrapersonal motivation, change, or alleviation of physical limitations [13,31]. Furthermore, if elderly people can engage in group activities that provide them with opportunities to socialize, there is a high possibility that they will continue to engage in health-promoting behaviors [22].

At the community level, given the nature of the rural regions, elderly people who consider farming as their job prioritized farming over health-promoting behaviors and did not consider doing both. Furthermore, it must also be taken into account that the sample consisted of elderly women, who not only farm, but also do house chores, which are not recognized as physical activities. A research that analyzed the attitudes towards health promotion among elderly Austrians [22] categorized house chores and walking that can help maintain health as a type of health promotion, a term coined “health promotion through daily activities”.

There are important differences between rural and urban areas, including regional factors, such as public services and physical environments [13,16,30,32]. In rural regions, diverse community resources are lacking and accessibility issues must be addressed for people to be able to participate in the programs that are being implemented [32]. It has been reported that elderly people in rural areas cannot participate in health-promoting activities because of public transportation issues, unsafe environments due to nearby crimes, uneven pavement, and environmental barriers that make access to facilities difficult [22]. If access to health facilities is difficult, elderly people would likely not participate in health programs. Alternatively, if elderly people are aware of the community resources but do not use them, this might be due to bad weather, insufficient time, lack of transportation, or costs. As a result, decentralization of facilities and programs is also recommended, especially in rural regions with infrequent public transportation options [33]. In addition, it is suggested that health- promoting programs that provide social opportunities without focusing on health can help the elderly maintain a healthy lifestyle [22].

In terms of public policy factors, the participants in this study mentioned insufficient provision of medical products. Previous research has also reported that inability to buy necessary medical products was a policy barrier to the access to healthcare services for the elderly in vulnerable regions in the United States [16]. From the perspective of public policy, strengthening the socio-economic capabilities of local organizations and improving the physical environments, such as parks, can reduce the risk of obesity among the residents and help to prevent stress [13]. A community-centered, health-promoting strategy can be effective to promote healthy lifestyles in people who lack socioeconomic resources and opportunities and have weak social support base, such as residents of rural areas [34]. This can be enacted through national or local policies. Improving the physical environment and providing health resources equitably through policies for the improvement of community residents’ health and quality of life, such as sports vouchers, seniors’ health education [31], or health city exercises, can increase the likelihood of residents’ engagement in health-promoting activities [13].

The study results showed that the multi-level factors proposed by the ecological theory interact with each other. However, among the barriers to health-promoting behaviors, a substantial number is concentrated in the intrapersonal factors; participants mentioned interpersonal, community, and public policy factors less frequently. This implies a lack of health-promoting policies or the insufficient resources allocated to health services in rural regions. Alternatively, it shows the possibility that the participants are not even aware of the ineffective health promotion public policies in the community.

Additionally, our findings reveal the rural elderly women’s lack of understanding of the concept of health promotion. While talking about health-promoting activities and their engagement in managing and improving their health, they focused mainly on exercises and physical activities. Through this study, it was shown that relevant factors and the barriers perceived by the elderly must be closely reviewed at multiple levels when implementing health-promoting activities for elderly people in vulnerable regions, such as rural regions. Furthermore, it is necessary to create conditions for active health education by utilizing health care officials at health clinics located in rural regions so that there are no information gaps due to regional differences [35].

This study has some limitations that should be considered when interpreting the results. The fact that this study was conducted in one particular rural region should be taken into consideration when applying the results to different cultures or contexts. Furthermore, it is possible that elderly people with poorer health conditions or who are more isolated due to mental problems were not included as participants in these focus group interviews. Therefore, it is important to conduct this research on a larger scale in the future. Despite these shortcomings, the present research distinguished and identified rural elderly women’s perceived barriers to health-promoting behaviors on a multi-level basis. Thus, this study can help meet the needs of local health policies by providing data useful to develop and implement customized health-promoting programs.

## 5. Conclusions

This study conducted an in-depth exploration of the perceived barriers to health-promoting behaviors among rural elderly women from an ecological perspective. The most common barriers were associated with intrapersonal factors. Depending on each individual’s health condition, health-promoting activities can be practiced at various levels of intensity. As intrapersonal and interpersonal factors affect each other to reduce or eliminate the perceived barriers to health-promoting behaviors, interventions addressing them could lead to desirable health-promoting activities. In particular, socialization of the rural elderly people can encourage health-promoting activities. In addition, a psychological approach aimed at developing a customized health promotion program reflecting the physical functional status of elderly women in rural areas or at changing their passive attitude into an active one may be useful. Moreover, a step-by-step approach may be necessary to actually practice health-promoting activities. From the community and public policy perspectives, it is necessary to provide integrated services based on programs that can foster desirable perceptions and attitudes about health promotion and strategies that can promote the socialization of elderly people.

## Figures and Tables

**Table 1:** Focus group interview questions.

Type of Question	Application	Question
Opening questions	We started with an easy question so that the participants could engage in the interview in a comfortable state.	- Can you introduce yourselves?
Introductory questions	We let the participants know about the general direction of the interview topic and let them speak their mind naturally.	- How do you think your health is?
Transition questions	We asked leading questions so that participants knew about the specific subject within a wide-ranging topic.	- What activities do you practice for your health?
Key questions	As these questions constitute the core of the research and are the primary focus of analysis, we allocated sufficient time so that the participants could thoroughly talk about their thoughts and experiences.	- If you do not think that you are able to engage in health-promoting behaviors, what do you think the reason is? - What do you think is the reason for not being able to engage in health- promoting behaviors for yourself? (intrapersonal) - What do you think is the reason for not being able to engage in health- promoting behaviors in relation to other people, such as family or neighbors? (interpersonal) - What is inconvenient about where you live when it comes to engaging in health-promoting behaviors? (community) - If you do not think that you are able to engage in health-promoting behaviors, what do you think is the reason in relation to the national or local government? (public policy)
Ending questions	At the end of the group interview, we asked the participants whether there was anything to add.	- Is there anything you want to say for the last time? - Is there anything missing or anything to add related to the topic we talked about today?

**Table 2:** General characteristics of the research participants (*N* = 26).

Variables		Mean ± SD (Range) or *N* (%)
Age (years)		78.81 ± 7.34 (65–91)
Family arrangement	Spouse	11 (42.3)
	Son	7 (26.9)
	Daughter	1 (3.8)
	Alone	7 (26.9)
Education	Never attended school	14 (53.8)
	Elementary school	8 (30.8)
	Middle school	4 (15.4)
Number of chronic diseases	1	2 (7.7)
	2	19 (73.1)
	3	5 (19.2)
